# Diabetes and Hemoglobin A1c as Risk Factors for Nosocomial Infections in Critically Ill Patients

**DOI:** 10.1155/2013/279479

**Published:** 2013-12-29

**Authors:** Eirini Tsakiridou, Demosthenes Makris, Vasiliki Chatzipantazi, Odysseas Vlachos, Grigorios Xidopoulos, Olympia Charalampidou, Georgios Moraitis, Epameinondas Zakynthinos

**Affiliations:** ^1^Department of Critical Care Medicine, University Hospital of Larissa, University of Thessaly School of Medicine, Biopolis, GR41000 Larisa, Greece; ^2^Department of Critical Care Medicine, General Hospital of Serres, GR62100 Serres, Greece; ^3^Department of Hematology Laboratory, General Hospital of Serres, GR62100 Serres, Greece

## Abstract

*Objective*. To evaluate whether diabetes mellitus (DM) and hemoglobin A1c (HbA1c) are risk factors for ventilator-associated pneumonia (VAP) and bloodstream infections (BSI) in critically ill patients. *Methods*. Prospective observational study; patients were recruited from the intensive care unit (ICU) of a general district hospital between 2010 and 2012. Inclusion criteria: ICU hospitalization >72 hours and mechanical ventilation >48 hours. HbA1c was calculated for all participants. DM, HbA1c, and other clinical and laboratory parameters were assessed as risk factors for VAP or BSI in ICU. *Results*. The overall ICU incidence of VAP and BSI was 26% and 30%, respectively. Enteral feeding OR (95%CI) 6.20 (1.91–20.17; *P* = 0.002) and blood transfusion 3.33 (1.23–9.02; *P* = 0.018) were independent risk factors for VAP. BSI in ICU (*P* = 0.044) and ICU mortality (*P* = 0.038) were significantly increased in diabetics. Independent risk factors for BSI in ICU included BSI on admission 2.45 (1.14–5.29; *P* = 0.022) and stroke on admission2.77 (1.12–6.88; *P* = 0.029). Sepsis 3.34 (1.47–7.58; *P* = 0.004) and parenteral feeding 6.29 (1.59–24.83; *P* = 0.009) were independently associated with ICU mortality. HbA1c ≥ 8.1% presented a significant diagnostic performance in diagnosing repeated BSI in ICU. *Conclusion*. DM and HbA1c were not associated with increased VAP or BSI frequency. HbA1c was associated with repeated BSI episodes in the ICU.

## 1. Introduction

Ventilator-associated pneumonia (VAP) often complicates the clinical course of the critically ill patients and it may be associated with considerable morbidity and mortality [1]. VAP can be precipitated by several factors which may be associated with the underlying disease, the critical interventions that are performed in the intensive care unit (ICU), or the logical impairment that has been described in the critical illness [2, 3].

Hyperglycemia and diabetes mellitus (DM) are conditions which have been linked to alterations of immune response and are often encountered in the critical care setting [4]. However, acute hyperglycemia is usually treated in ICU and the level of alertness of physicians for the possible complications of acute hyperglycemia and DM in critically ill patients is high [5]. On the other hand, it is not known whether diabetic patients with a history of less optimum diabetic control, such as those with abnormal hemoglobin A1c (HbA1c), could be at a higher risk of ICU infections compared to diabetics with a history of well-controlled DM prior to ICU admission. Previous studies have examined the correlation between HbA1c and the outcome in diabetic patients with sepsis but the association between HbA1c and VAP is not clear [6, 7].

In this respect, we hypothesized that DM and HbA1c, which are an index of overall glycemic exposure relatively unaffected by acute changes (e.g., stress, illness), might be associated with nosocomial infections which are most commonly encountered in the ICU such as VAP or bloodstream infections (BSI). We, therefore, aimed to assess nosocomial infections in the ICU patients prospectively and to investigate whether DM and increased HbA1c levels are independent risk factors for VAP and BSI [8].

## 2. Methods

The present prospective observational study was conducted in a general district hospital in Greece between 2010 and 2012. Consecutive sampling was used to recruit patients admitted to the general ICU of the hospital. The study was approved by the University of Thessaly (UT 08-02-10/568) and the International Centre of Services of Health Ministry (UT 01-06-10/2124).

Inclusion criteria were (a) intubation for more than 48 hours and (b) ICU hospitalization for more than 72 hours. Exclusion criteria were (a) presence of hemoglobinopathies, (b) blood transfusion prior to ICU admission with 2 or more RBCs units [8], (c) white blood cells < 0.5 × 10/L, or (d) malignancy under chemotherapy.

### 2.1. Study Groups

Patients were classified based on their medical history as diabetics and nondiabetics. Diabetics were further classified as having normal glycemic control (NGC) or abnormal glycemic control (AGC) and if they had HbA1c < 7% or HbA1c ≥ 7%, respectively, according to American Diabetes Association (ADA) guidelines [9]. Patients without a history of diabetes and HbA1c ≥ 7% were also considered as diabetics with AGC.

### 2.2. Outcomes

The main outcome evaluated was the relationship between HbA1c levels and VAP or BSI. Secondly, we assessed the relationship between HbA1c and ICU mortality.

### 2.3. Clinical Evaluation

Baseline demographic data including age, sex, body mass index (BMI), and medical history including APACHE II score, previous antibiotic exposure, previous hospitalizations, and corticosteroids exposure were recorded. All patients admitted to the ICU, except those that did not meet criteria for entry in analysis, were then followed until hospital discharge for early or late onset VAP, BSI, other types of infection, and sepsis. Clinical pulmonary infection score (CPIS) was evaluated as reported [10]. Potential risk factors for VAP and BSI were recorded during ICU stay; risk factors were required to be present at least 48 hours before VAP or BSI diagnosis.

### 2.4. HbA1c Measurement and Sample Collection

A whole blood glucose specimen was taken during the first 24 hours of ICU admission for HbA1c measurement. HbA1c was measured by Cobas Integra 400 Plus TQ HbA1c Gen. 2 (Roche Diagnostics Ltd. Switzerland/Windows XP) which is an immunoassay method for the quantitative determination of percent HbA1c and the laboratory range of HbA1c was 4.4–7%. Blood, urine, and tracheal aspirates with a sterile sputum trap for semiquantitative cultures were collected routinely on ICU admission but also during the hospital stay whenever there were clinical and laboratory signs of infection [11].

### 2.5. Definitions

VAP was defined as a new persistent chest-radiographic infiltrate in conjunction with one of the following: a positive blood or pleural fluid culture or two of the following: fever (temperature > 38.3°C), leukocytosis (leukocyte count > 10^4^/mm^3^), and purulent tracheal aspirate [1]. In addition, a positive tracheal aspirate culture was required to confirm the diagnosis of VAP [10]. BSI was defined according to CDC/NHSN definition of healthcare-associated infections [12]. Sepsis was defined as previously suggested by international consensus guidelines [13]. Multidrug-resistant (MDR) bacteria was defined as follows: methicillin resistant *Staphylococcus aureus*, ceftazidime or imipenem resistant *Pseudomonas aeruginosa*, colistin sensitive *Acinetobacter baummannii* and *Stenotrophomonas maltophilia*, and gram-negative bacilli producing extended spectrum beta lactamase [14].

### 2.6. Statistics

Data are presented as frequency (%) for qualitative parameters or mean ± SD for quantitative variables. Comparisons between groups were performed by using *t*-test/ManWhitney-*U* tests or chi-square/Fishers exact test as appropriate. ROC curves were constructed to investigate the diagnostic performance of HbA1c in diagnosing VAP or BSI. Univariate analysis was performed to determine variables potentially associated with VAP, BSI, and ICU mortality. For VAP and BSI, variant factors such as demographics, DM, HbA1c ≥ 7%, comorbidities, corticosteroids and blood transfusion in ICU, previous antibiotic and corticosteroid exposure, previous surgery, previous infection, and enteral or parenteral feeding were all considered. Factors affecting mortality included demographics, DM, HbA1c ≥ 7%, Apache II score, comorbidities, previous hospitalization, previous surgery, previous infection, enteral or parenteral feeding, ICU-aquired infections, and sepsis. Variables which were significant at the 0.05 level were then included in multivariate logistic regression analysis. Model's calibration was tested using the Hosmer-Lemeshow goodness-of-fit statistic. A high *P* value (0.05) would indicate a good fit for the model. The overall accuracy (discrimination) of the model has been evaluated too. Data were analyzed using SPSS version 17.

## 3. Results

**Figure 1:**
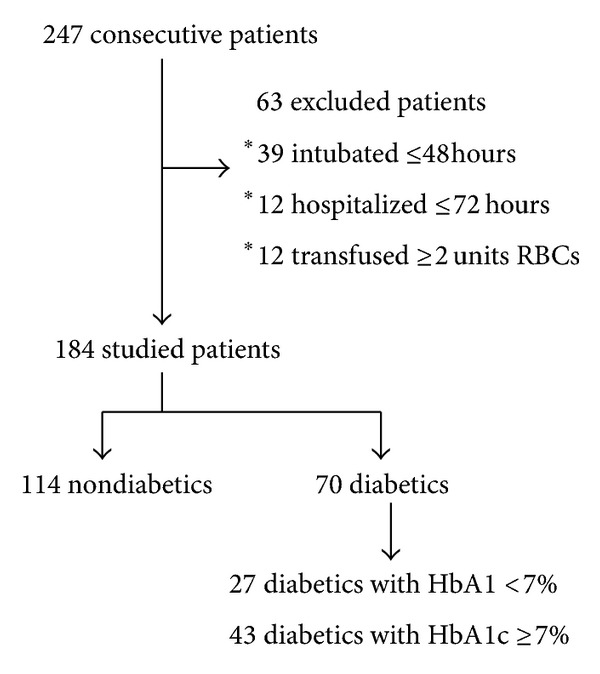
Flow chart of the study.

One hundred and eighty-four patients out of 247 admissions were eligible and entered the study. Sixty-three patients were excluded, 51 patients were intubated <48 hours or hospitalized <72 hours, and 12 patients had received ≥2 units of RBCs prior to HbA1c analysis. There were 70 diabetics: 43 (61%) were classified as AGC and 27 (39%) as NGC (Figure 1). There were no statistical differences between the 3 analyzed groups in respect to the cause of admission (Table 1). Diabetic patients were significantly older (*P* = 0.001) and more obese (*P* = 0.026), they had more elevated glucose on admission (*P* < 0.001) and Apache II score (<0.001) and higher HbA1c (*P* < 0.001), and presented significantly higher percentage of cardiac disease (*P* < 0.001), hypertension (*P* = 0.002), and chronic kidney disease (*P* = 0.008) in their medical history compared to nondiabetics.

### 3.1. Ventilator Associated Pneumonia

VAP was diagnosed in 47 patients (26%). In the majority of cases, the cause of admission was respiratory failure (38%) or neurologic disorders (23%). The frequency of VAP was similar among the study groups. The mean time for VAP diagnosis was 12 ± 8 days.

The pathogens associated with VAP for all patients are shown in Table 2. The most frequent microorganism causing VAP was *Acinetobacter baummanni* (39%). NGC patients and nondiabetics presented significantly more frequent gram-negative MDR bacterial VAP compared to patients with AGC; AGC patients presented more frequently gram-positive bacterial VAP (*P* = 0.02).

BSIs on admission (OR (95% CI) 2.218 (1.023–4.808), *P* = 0.041) or in ICU (95% CI) 2.892 (1.267–6.597), *P* = 0.009, enteral (95% CI) 6.474 (3.020–13.881), *P* < 0.001, or parenteral feeding (95% CI) 7.892 (2.683–23.221), *P* < 0.001, and blood transfusion (95% CI) 3.746 (1.727–8.127), *P* = 0.001, were significantly associated with the presence of VAP, whereas HbA1c ≥ 7% was not (*P* = 0.685) (Table 3). After excluding patients with tracheostomy on admission and those who developed VAP before tracheostomy, the incidence of VAP in those who had a tracheostomy in the ICU was 65% whereas for those who had not undergone tracheostomy was 36% (95% CI) 3.250 (1.434–7.362) *P* = 0.004. Multivariate analysis showed that enteral feeding (95% CI) 6.205 (1.909–20.170), *P* = 0.002, and blood transfusion (95% CI) 3.327 (1.227–9.022), *P* = 0.018, were significantly associated with VAP (Table 4). Furthermore, multivariate analysis showed that enteral feeding (95% CI) 3.728 (1.281–10.848), *P* = 0.016, and respiratory tract infection on admission (95% CI) 3.691 (1.328–10.256), *P* = 0.012, were the only independent risk factors for developing MDR-VAP.

### 3.2. Bloodstream Infections

BSI was diagnosed in 55 (30%) patients. In the majority of cases it concerned patients with respiratory failure (40%) and neurologic disorders (20%). BSI was significantly increased in diabetics (39% versus 25% *P* = 0.044). In addition, NGC patients presented significantly increased incidence of BSI caused by MDR bacteria compared to AGC patients (22% versus 5% *P* = 0.025).

Risk factors for BSI were DM (95% CI) 1.929 (1.014–3.669; *P* = 0.044), BSI on admission (95% CI) 2.590 (1.222–5.489; *P* = 0.011), and the medical history of stroke (95% CI) 2.994 (1.230–7.286; *P* = 0.013) (Table 5). However in a multiple regression analysis, BSI on admission (95% CI) 2.452 (1.136–5.295; *P* = 0.022) and medical history of stroke (95% CI) 2.768 (1.112–6.885; *P* = 0.029) were identified as the only independent risk factors for BSI in ICU (Table 6).

When ROC curve analysis was applied to examine whether there is any correlation between levels of HbA1c and the incidence of BSI in those patients who had more than one episode of BSI during their ICU stay, analysis showed that the diagnostic performance of HbA1c in the diagnosis of repeated episodes of BSI (>one episode) in ACG patients was significant (HbA1c ≥ 8.1%, sensitivity, specificity 100%, and 65%, resp., AUC = 0.850, *P* = 0.04).

### 3.3. Secondary Outcomes

The mean ICU stay was 16 ± 15 days; ICU stay was similar among different groups. Overall, ICU mortality was significantly higher in diabetics compared to nondiabetics (95% CI) 1.953 (1.033–3.693; *P* = 0.038). Risk factors for death in all analyzed patients were age (71 ± 12 versus 59 ± 18 *P* < 0.001), Apache II score (26 ± 7 versus 21 ± 7; *P* < 0.001), previous hospitalization (95% CI) 1.964 (1.052–3.666; *P* = 0.033), DM (OR (95% CI) 1.953 (1.033–3.693; *P* = 0.038), hypertension (95% CI) 2.044 (1.117–3.741; *P* = 0.02), cardiac disease (95% CI) 2.018 (1.097–3.710; *P* = 0.023), chronic kidney disease (95% CI) 4.760 (1.048–21.618; *P* = 0.028), parenteral feeding (95% CI) 5.127 (1.564–16.807; *P* = 0.003), and the development of sepsis (95% CI) 2.544 (1.302–4.971; *P* = 0.006). Multivariate analysis showed that sepsis (95% CI) 3.336 (1.468–7.580; *P* = 0.004) and parenteral feeding (95% CI) 6.295 (1.596–24.826; *P* = 0.009) were independently associated with ICU mortality. For the selected model, the Hosmer-Lemeshow chi-square = 6.327, df = 8, *P* = 0.611 was not significant denoting that it was well calibrated and fitted the data well. The overall accuracy of the model was 74.5%.

There were no other differences between diabetics and nondiabetics, between nondiabetics and AGC patients, or between NGC and AGC patients regarding other types of infection, such as urinary tract infection, catheter related infection, and sepsis.

## 4. Discussion

Our findings do not support the hypothesis that HbA1c is associated with increased risk of VAP or BSI in the ICU. To our knowledge, this is the first study to examine such a relationship. However, a significant association between HbA1c and BSI was found in this study, while a threshold of HbA1c ≥ 8.1% was found to be significant in the diagnosis of repeating an episode of BSI among patients with uncontrolled diabetes.

In the present study there was no significant relationship between DM or HbA1c and VAP. According to the literature, hyperglycemia in diabetics predisposes to infections because of the impairment of innate cellular immunity, especially in diabetics with poor glycemic control [4, 15, 16]. Moreover, critically ill patients independent of DM are prone to stress-induced hyperglycemia which increases the risk of complications and leads to an adverse outcome [17]. Therefore, one could expect that critically ill diabetics and particularly those with increased HbA1c would be more susceptible to ICU infections including VAP, compared to nondiabetics and diabetics with adequate glucose control, respectively. However, there are also data that support that the risk of nosocomial pneumonia in DM patients treated in the ward or in the ICU is not significantly increased [18]. This is in agreement with the findings of the present study. The most plausible explanation could be that a combination of factors is required so as to result in clinically significant respiratory infection in the ICU, such as microaspirations, increased severity of illness, and duration of mechanical ventilation, and that diabetic immune dysfunction is not sufficient enough, therefore, to increase VAP frequency.

Nevertheless, our findings provide evidence that DM might be significantly associated with the incidence of BSI in the ICU. DM was identified as a risk factor for BSI. In addition, patients with increased HbA1c were at significant risk of repeated BSI and a cut-off level of ≥8.1% presented significant diagnostic performance in diagnosing repeated BSI. This association between DM and BSI has been underlined previously [19]. Our findings point out the necessity of BSI control measures in the ICU, especially in diabetic patients.

In the present study, we evaluated several risk factors for increased frequency of VAP. VAP was related to enteral feeding and blood transfusion which have been previously suggested as risk factors for VAP. Enteral nutrition has been indicated as an independent risk factor for VAP from several existing studies, since it might increase the risk of aspiration of gastric contents [20, 21]. Several preventive measures were implemented to minimize the gastric reflux of all treated patients. However, some factors related to enteral feeding, such as the continuous control of cuff pressure or the mode of feeding (continuous or intermittent), have not been adequately controlled and this might have favored microaspiration in the lungs [22, 23].

Blood transfusion was also a risk factor for VAP. This relationship has been reported in previous studies [24, 25]. It is believed that the transfusion of nonleukocyte-depleted red blood cell units due to the biochemical changes occurring during blood storage may have immunosuppressive effects [26]. Thus, routine red blood transfusion should be conducted with a restricted transfusion trigger policy as it has been previously recommended [11].

On the other hand, in contrast to previous studies, tracheostomy was not identified as an independent risk factor for VAP [21, 27]. Based on the pathophysiology of VAP, tracheotomized patients are probably at decreased risk of VAP. Several factors support this hypothesis. Tracheostomy reduces the risk of inhalation of oropharyngeal secretions by liberating the vocal cords and facilitates weaning from mechanical ventilation [28]. A possible explanation of this finding by “Nseir and coworkers”, who found that tracheostomy was independently associated with decreased risk of VAP, is that tracheostomy is rather a marker of longer duration of mechanical ventilation than a risk factor for VAP [29].

DM and AGC were not associated with VAP due to MDR. NGC diabetic patients and nondiabetics presented significantly increased incidence of VAP due to MDR than AGC diabetics. However, MDR-VAP was not independently associated with known risk factors such as previous antibiotic therapy and previous hospitalization [11]. In this study, enteral feeding and respiratory tract infection on admission were the only independent risk factors for MDR-VAP. This finding could be ascribed to previous increased sensitivity of the respiratory system or may indicate an ICU exposure to broad-spectrum antibiotic treatment which is a known risk factor for MDR acquired infections. On the other hand, enteral feeding may predispose more frequently to aspiration of gastric material where gram-negative germes predominate [22].

BSI in ICU was significantly associated with the presence of DM. In contrast to other similar studies, only BSI on admission and a history of stroke were independently associated with BSI [19]. A possible interpretation of this finding could be that these particular risk factors may point out a previous immunosuppression of patients developing BSI, due to possible complications which arise from stroke leading to malnutrition, impaired hygiene, and infections [30].

Diabetic patients presented significantly increased mortality than nondiabetics in this study. ICU mortality was independently associated with factors that have been known to affect ICU mortality such as sepsis, age, and parenteral feeding, but DM was not an independent risk factor for mortality. ICU morality is multifactorial and the impact of DM in mortality might be not straight forward. Previous studies showed that mortality in critically ill patients has been found to be related to DM only in cardiac surgery patients or in patients with acute organ failure or septicemia [31, 32]; yet, recent studies in mixed ICUs showed that critically ill patients with DM did not have an increased mortality compared to nondiabetic patients [33, 34].

In this study, we found that parenteral feeding was associated with increased mortality. Parenteral feeding has been associated with higher risks of intravascular catheter related infections and metabolic disturbances such as hyperglycemia and enteral microbial translocation because of the loss of intestinal villous architecture. The fact that this may potentially lead to a poor outcome is a matter of conflict [35, 36]. In this respect, our study rather emphasizes the need for adopting measures for the optimum time of initiating parenteral feeding and for the prevention of colonization of central venous catheters [37].

There are some points that have to be taken into consideration when interpreting the results of the present study. First, patients with hemoglobinopathies and blood transfusion patients were excluded from the study. This was due to the possible interference of the hemoglobin trait with the assay method and because, according to NGSP recommendations, any condition that changes red cell turnover will falsely lower HbA1c test results regardless of the assay method used [8]. This methodology has also been used in previous studies [38]. Secondly, diagnosis of diabetes in critically ill patients was based on their medical history and levels of abnormal glucose control HbA1c ≥ 7% for patients without history of DM, since diagnostic test for diabetes, such as fasting glucose levels and oral glucose tolerance, might be not available during acute critical illness. According to the latest ADA recommendations, diagnosis of diabetes can be made if HbA1c level is ≥6.5% and diagnosis should be confirmed by repeating testing in the absence of unequivocal hyperglycemia. Nevertheless, taking into account that stress-induced hyperglycemia is common in critically ill patients, it was difficult to characterize patients without known diabetes as diabetics, unless they had HbA1c ≥ 7%. Thus, the possibility of misclassified diabetic patients with unknown diabetes and normal glucose control (HbA1c 6.5%–7%) as nondiabetics cannot be excluded. However, there were only six of such cases. Finally, tracheal aspirates were examined by the use of semiquantitative cultures which may be less accurate for the diagnosis of VAP; however, we used the generally accepted definition of ATS for VAP diagnosis where semiquantitive cultures can be also used for diagnostic purposes [11].

In conclusion, our study suggests that DM and glucose regulation before the ICU admission might be not related to VAP. However, our data also showed that increased HbA1c levels might be predictive of increased risk for repeating BSI in the ICU. In this respect, HbA1c might be useful as a diagnostic biomarker in critical care patients.

## Figures and Tables

**Table 1:** Baseline characteristics of all study groups as determined by hemoglobin A1c (HbA1c) and history.

	Nondiabetics *n* = 114	Diabetics *n* = 70	Diabetics with AGC *n* = 43	Diabetics with NGC *n* = 27
Sex (male)	70 (61%)	38 (54%)	26 (61%)	12 (44%)
Age (years)	63 ± 18	71 ± 10*	70 ± 11	73 ± 8
Apache II score	23 ± 8	27 ± 6*	26 ± 6	28 ± 6
*Β*M*Ι*	28 ± 7*	30 ± 6	30 ± 5	30 ± 7
HbA1c (%)	5,9 ± 0,4	7,7 ± 1,6*	8,5 ± 1,6**	6,5 ± 0,3
Initial glucose (mg/dL)	168 ± 71	248 ± 98*	263 ± 107	223 ± 77
Glucose of exit	136 ± 60	146 ± 68	149 ± 70	142 ± 65
Comorbidities				
Hypertension	51 (45%)	48 (69%)*	28 (65%)	20 (74%)
Hyperlipidemia	20 (18%)	23 (33%)	15 (35%)	8 (30%)
Cardiac disease	40 (35%)	48 (69%)*	30 (70%)	18 (67%)
Stroke	12 (11%)	11 (16%)	5 (12%)	6 (22%)
Chronic kidney disease	5 (4%)	11 (16%)*	6 (14%)	5 (19%)
Cancer	11 (10%)	2 (3%)	2 (5%)	0%
COPD	22 (19%)	15 (21%)	11 (26%)	4 (15%)
Other	32 (28%)	18 (26%)	11 (26%)	7 (26%)
Category of admission				
Cardiac disorders	16 (14%)	18 (26%)	8 (30%)	10 (23%)
Neurologic disorders	23 (20%)	13 (19%)	3 (11%)	10 (23%)
Respiratory failure	42 (37%)	28 (40%)	10 (37%)	18 (42%)
Sepsis	2 (2%)	6 (9%)	3 (11%)	3 (7%)
*Τ*rauma	15 (13%)	2 (3%)	2 (7%)	0%
Surgical	9 (8%)	3 (4%)	1 (4%)	2 (5%)
Other	7 (6%)	0%	0%	0%

Data are presented as *n* (%) or mean (±SD). Differences between the analyzed groups of the study were evaluated by *t*-test or *χ* ^2^ test as appropriate.

BMI: body mass index, COPD: chronic obstructive pulmonary disease, NGC: normal glucose control, AGC: abnormal glucose control.

*P < 0.05 between diabetics and nondiabetics.

**P < 0.05 between diabetics with AGC and diabetics with NGC.

**Table 2:** Microbiology of ventilator-associated pneumonia (VAP) including a second episode in each analyzed group of the study.

	Nondiabetics *n* = 32	Diabetics with AGC *n* = 12	Diabetics with NGC *n* = 10
*Pseudomonas aeruginosa *	6 (19%)	1 (8%)	2 (20%)
*Klebsiella spp. *	4 (13%)	1 (8%)	1 (10%)
*Enterobacter spp. *	4 (13%)	1 (8%)	0
*Proteus spp. *	2 (6%)	1 (8%)	0
*Acinetobacter baummannii *	13 (41%)	3 (25%)	5 (50%)
*Stenotrophomonas maltophilia *	1 (3%)	0	1 (10%)
*Morganella morganii *	0	0	1 (10%)
Total gram-negative bacteria	30 (94%)*	**7 (58%)**	10 (100%)**
CNS	1 (3%)*	3 (25%)	0
MSSA	1 (3%)	0	0
*Enterococcus faecium *	0*	2 (17%)	0**
Total gram-positive bacteria	2 (6%)*	**5 (42%)**	0**

Data are presented as *n* (%).

CNS: coagulase negative Staphylococci, MSSA: methicillin sensitive Staphylococcus aureus, NGC: normal glucose control, AGC: abnormal glucose control.

*P < 0.05 between diabetics with AGC and nondiabetics.

**P < 0.05 between diabetics with AGC and diabetics with NGC.

**Table 3:** Risk factors for ventilator-associated pneumonia (VAP).

	Patients with VAP *n* = 47	Patients without VAP *n* = 137	OR	95% CI	*P *
Age (years)	65 ± 14	66 ±17			0.568
*Β*MI	28 ± 5	28 ± 8			0.956
Apache II score	24 ± 5	25 ± 8			0.733
Sex (male)	29 (62%)	79 (58%)	1.183	0.600–2.332	0.628
Diabetes Mellitus	20 (43%)	50 (37%)	1.289	0.656–2.531	0.461
HbA1c ≥ 7%	12 (26%)	31 (23%)	1.172	0.544–2.527	0.685
Hypertension	20 (43%)	79 (58%)	0.544	0.278–1.063	0.073
Hyperlipidemia	9 (19%)	34 (25%)	0.717	0.315–1.635	0.428
Cardiac disease	15 (32%)	73 (53%)	0.411	0.204–0.827	0.011
COPD	7 (15%)	30 (22%)	0.624	0.254–1.534	0.301
Chronic kidney disease	4 (9%)	12 (9%)	0.969	0.297–3.164	0.958
Stroke	5 (11%)	18 (13%)	0.787	0.275–2.252	0.655
Cancer	3 (6%)	10 (7%)	0.866	0.228–3.290	0.832
Other	12 (26)	38 (28%)	0.893	0.420–1.900	0.769
Enteral feeding	36 (77%)	46 (34%)	6.474	3.020–13.881	<0.001
Parenteral feeding	43 (92%)	79 (58%)	7.892	2.683–23.221	<0.001
Blood transfusion in ICU	17 (36%)	18 (13%)	3.746	1.727–8.127	0.001
Corticosteroids in ICU	17 (36%)	30 (22%)	2.021	0.984–4.151	0.053
BSI in ICU	13 (28%)	16 (12%)	2.892	1.267–6.597	0.009
Infection on admission	22 (47%)	50 (37%)	1.531	0.783–2.993	0.211
BSI on admission	14 (30%)	22 (16%)	2.218	1.023–4.808	0.041
Previous surgery	7 (15%)	18 (13%)	1.152	0.450–2.973	0.762
Preceding intake of antibiotics	23 (49%)	52 (38%)	1.567	0.803–3.055	0.186
Preceding corticosteroid treatment	1 (2%)	7 (5%)	0.404	0.048–3.371	0.387
Previous hospitalization	21 (45%)	55 (40%)	1.204	0.617–2.351	0.586

Data are presented as *n* (%) or mean (±SD).

VAP: ventilator-associated pneumonia, ICU: intensive care unit, BMI: body mass index, COPD: chronic obstructive pulmonary disease, BSI: bloodstream infections.

**Table 4:** Multivariate analysis for risk factors for ventilator-associated pneumonia (VAP) in all analyzed patients.

	OR	95% CI	Wald statistic	*P *
BSI on admission	2.557	0.823–7.942	2.637	0.104
BSI in ICU	1.229	0.412–3.667	0.137	0.712
Blood transfusion in ICU	3.327	1.227–9.022	5.576	0.018
Enteral feeding	6.205	1.909–20.170	9.210	0.002
Parenteral feeding	2.100	0.493–8.953	1.006	0.316
Tracheostomy	1.353	0.507–3.610	0.366	0.545

The Hosmer-Lemeshow statistic (calibration) was not significant: *χ* ^2^ = 9.667, df = 7, *P* = 0.208. Overall accuracy (discrimination), 85.8%. BSI: bloodstream infections, ICU: intensive care unit.

**Table 5:** Risk factors for bloodstream infections (BSI)

	Patients with BSI *n* = 55	Patients without BSI *n* = 129	OR	95% CI	*P *
Age (years)	66 ± 15	66 ± 16			0.845
*Β*MI	29 ± 5	28 ± 8			0.789
Apache II score	25 ± 7	24 ± 8			0.746
Sex (male)	37 (67%)	71 (55%)	1.679	0.867–3.254	0.123
Diabetes Mellitus	27 (49%)	43 (33%)	1.929	1.014–3.669	0.044
HbA1c ≥ 7%	15 (27%)	28 (22%)	1.353	0.654–2.796	0.414
Hypertension	30 (55%)	69 (54%)	1.043	0.544–1.966	0.895
Hyperlipidemia	10 (18%)	33 (26%)	0.646	0.293–1.426	0.278
Cardiac disease	23 (42%)	65 (50%)	0.708	0.374–1.339	0.287
COPD	12 (22%)	25 (19%)	1.161	0.535–2.519	0.706
Chronic kidney disease	4 (7%)	12 (9%)	0.765	0.235–2.485	0.655
Stroke	12 (22%)	11 (9%)	2.944	1.230–7.286	0.013
Cancer	4 (7%)	9 (7%)	1.046	0.308–3.551	0.943
Other	16 (29)	34 (26%)	1.146	0.568–2.312	0.703
Enteral feeding	27 (49%)	64 (50%)	0.979	0.521–1.841	0.948
Parenteral feeding	48 (87%)	115 (89%)	0.835	0.317–2.197	0.714
Blood transfusion in ICU	13 (24%)	26 (20%)	1.226	0.576–2.612	0.597
Corticosteroids in ICU	15 (27%)	35 (72%)	1.007	0.496–2.047	0.984
Previous infection in ICU	17 (31%)	26 (20%)	1.772	0.866–3.625	0.115
Infection on admission	23 (42%)	49 (38%)	1.173	0.617–2.232	0.626
BSI on admission	17 (31%)	19 (15%)	2.590	1.22–5.489	0.011
Previous surgery	5 (9%)	20 (16%)	0.545	0.193–1.535	0.245
Preceding intake of antibiotics	21 (38%)	54 (42%)	0.858	0.449–1.638	0.642
Preceding corticosteroid treatment	4 (7%)	4 (3%)	2.451	0.590–10.177	0.204
Previous hospitalization	23 (42%)	53 (41%)	1.031	0.543–1.955	0.926

Data are presented as *n* (%) or mean (±SD).

ICU: intensive care unit, BMI: body mass index, COPD: chronic obstructive pulmonary disease, BSI: bloodstream infections.

**Table 6:** Multivariate analysis for risk factors for bloodstream infections (BSI) in all analyzed patients.

	OR	95% CI	Wald statistic	*P *
BSI on admission	2.452	1.136–5.295	5.217	0.022
Diabetes mellitus	1.802	0.928–3.501	3.026	0.082
Stroke	2.768	1.112–6.885	4.792	0.029

The Hosmer-Lemeshow statistic (calibration) was not significant: *χ* ^2^ = 9.446, df = 3, *P* = 0.124. Overall accuracy (discrimination), 75%. BSI: bloodstream infections.
